# In vivo variability of MRI radiomics features in prostate lesions assessed by a test-retest study with repositioning

**DOI:** 10.1038/s41598-025-09989-7

**Published:** 2025-08-13

**Authors:** Kevin Sun Zhang, Christian Jan Oliver Neelsen, Markus Wennmann, Thomas Hielscher, Balint Kovacs, Philip Alexander Glemser, Magdalena Görtz, Albrecht Stenzinger, Klaus H. Maier-Hein, Johannes Huber, Heinz-Peter Schlemmer, David Bonekamp

**Affiliations:** 1 German Cancer Research Center (DKFZ), Division of Radiology, Heidelberg, Germany; 2 German Cancer Research Center (DKFZ), Division of Biostatistics, Heidelberg, Germany; 3 German Cancer Research Center (DKFZ), Division of Medical Image Computing, Heidelberg, Germany; 4 Medical Faculty of Heidelberg University, Heidelberg, Germany; 5 Department of Urology, Heidelberg University Hospital, Heidelberg, Germany; 6 German Cancer Research Center (DKFZ), Junior Clinical Cooperation Unit ‘Multiparametric Methods for Early Detection of Prostate Cancer, Heidelberg, Germany; 7 Institute of Pathology, Heidelberg University Hospital, Heidelberg, Germany; 8 National Center for Tumor Diseases (NCT) Heidelberg, Heidelberg, Germany; 9 Pattern Analysis and Learning Group, Department of Radiation Oncology, Heidelberg University Hospital, Heidelberg, Germany; 10 Department of Radiology (E010), German Cancer Research Center (DKFZ), Im Neuenheimer Feld 280, 69120 Heidelberg, Germany

**Keywords:** Prostate, Magnetic resonance imaging, Reproducibility of results, Observer variation, Radiomics, Medical research, Biomarkers, Predictive markers

## Abstract

Despite academic success, radiomics-based machine learning algorithms have not reached clinical practice, partially due to limited repeatability/reproducibility. To address this issue, this work aims to identify a stable subset of radiomics features in prostate MRI for radiomics modelling. A prospective study was conducted in 43 patients who received a clinical MRI examination and a research exam with repetition of T2-weighted and two different diffusion-weighted imaging (DWI) sequences with repositioning in between. Radiomics feature (RF) extraction was performed from MRI segmentations accounting for intra-rater and inter-rater effects, and three different image normalization methods were compared. Stability of RFs was assessed using the concordance correlation coefficient (CCC) for different comparisons: rater effects, inter-scan (before and after repositioning) and inter-sequence (between the two diffusion-weighted sequences) variability. In total, only 64 out of 321 (~ 20%) extracted features demonstrated stability, defined as CCC ≥ 0.75 in all settings (5 high-b value, 7 ADC- and 52 T2-derived features). For DWI, primarily intensity-based features proved stable with no shape feature passing the CCC threshold. T2-weighted images possessed the largest number of stable features with multiple shape (7), intensity-based (7) and texture features (28). Z-score normalization for high-b value images and muscle-normalization for T2-weighted images were identified as suitable.

**Supplementary Information:**

The online version contains supplementary material available at 10.1038/s41598-025-09989-7.

## Introduction

Radiomics is a field of machine learning, in which pre-defined quantitative features are extracted from radiologic images, fed to learning algorithms to perform inference tasks like prediction or classification^1,2^. Especially in the field of multiparametric prostate MRI (mpMRI), radiomics has gained tremendous impact on research regarding prostate lesion classification (cancer vs. non-cancer)^3–5^. The interpretability of the pre-defined features is a major advantage compared to deep-learning based approaches^6,7^.

Despite great academic success, translation into the clinical setting has been lacking^8^. A large hindrance yet to overcome is the limited reproducibility of the features themselves, let alone the machine learning algorithms built upon extracted features^9^. Obvious factors to consider as sources for feature instability include imaging modality (e.g. CT vs. MRI), reconstruction method^10, ^scanner system^11^, magnetic field strength^11,12^, MRI sequence used^13, ^imaging artefacts, intra- and inter-reader variability^14,^ and even the tumor biology in oncologic imaging^15^. However, even when keeping all these factors identical, rescanning a patient twice on the same system will also result in variability due to different positioning and temporal fluctuations in the scanner setup^11,16^.

One approach to address feature instability is the separation of all available features into a group of reproducible and a group of non-reproducible (unstable) features, as proposed by different expert recommendations^8,9,17,18^. Repeatability/Stability of a radiomics feature is a basic requirement for its usefulness as an imaging biomarker^16^. Unstable features, which are identified to be vulnerable to acquisition and/or human reader effects, should be interpreted with caution. The identification of stable features allows reduction of the number of variables and focus on features with a higher potential to be biologically relevant. As radiomics feature extraction is always dependent on the definition of a region of interest (ROI), intra- and inter-rater variability should be part of the assessment^18,19^. Consequently, some studies do report on rater dependence as source of variability in general^19^ and in prostate mpMRI^20,21^, especially as this type of analysis can be performed post-hoc on already existing data. The study of in vivo test-retest variability, however, requires two scans of a patient within a short time frame and therefore a prospective study design which only few studies provide^11,16,22,23^. For optimal study of integrated retest and rater dependency, the most homogeneous setup regarding all other factors is provided by a single-scanner setting, with imaging before and after repositioning on the same day. This homogeneous approach allows to preserve the maximum amount of potentially useful features while identifying all features that are not even stable in the most homogeneous setting and therefore have limited potential to be useful^16,18^. The feature set reduction by the single-scanner setup is expected to be considerable^11,16^, while the approach preserves features which depend on between-scanner and between-institutional calibration. With other study designs, the difference between useless features and features requiring very homogeneous environments may not become apparent. As such, we believe that the homogeneous mono-centric establishment of a reliable radiomics features is a necessary foundation for efficient development and future multi-centric validation of radiomics feature sets. Thus, the homogeneous setup supports identification of the maximum potentially useful set of radiomics features, while the heterogeneous setup in multi-centric studies identifies the most robust set of features, however with the risk of removing useful features that require effort for inter-institutional calibration.

Therefore, the aim of this prospective study was to perform repeat acquisitions of T2- and diffusion-weighted imaging (T2WI / DWI) sequences during prostate MRI after patient re-positioning, and to derive and report a comprehensive set of potentially useful radiomics features for prostate MRI assessment.

## Patients and methods

### Study sample

The ethics committee of the medical faculty of Heidelberg University approved this prospective study (approval: S-059/2017) and all included patients provided informed consent. All experiments were performed in accordance with the relevant guidelines and regulations. Patients with clinical suspicion for clinically significant prostate cancer (sPC) due to elevated prostate specific antigen levels, abnormal digital rectal examination or due imaging in an active surveillance program were asked to participate in this study. Having agreed to the study, patients underwent regular prostate MRI (main scan) with the non-contrast sequences repeated (additional scan). The study was designed to test for the repeatability and reproducibility of quantitative and qualitative features in prostate lesions. The variability of one quantitative parameter - the mean of the apparent diffusion coefficient (ADC) - has been reported for this cohort previously^24^.

The inclusion criterion for the analysis was acquisition of both the main scan and the additional scan sequences in the MRI for at least one of the sequences. Exclusion criteria were: (i) past ablation / radiation therapy for prostate cancer, (ii) severe imaging artefacts, (iii) atypical histology upon biopsy (i.e., prostate tuberculosis, leiomoysarcoma), or (iv) an inconspicuous prostate without MRI-visible lesions.

### Imaging and MRI protocol

All patients were scheduled for a regular prostate MRI. Patients consenting to the study received a duplicate scan of the T2-weighted imaging (T2WI) and DWI sequences (additional scan) in addition to the regular (main) exam. Between the two scans, the patients were asked to exit the scanner, wait for 1–2 min and re-enter to finish the study.

All patients were examined on a 3T MRI (Siemens Magnetom Prisma, Siemens Healthcare, Erlangen/Germany) using standard 18-channel body and integrated spine phasedarray receiver coils. The three axial sequences used for the study were (i) T2-weighted turbo spin echo (TR 3710–9370 ms, TE 96–145 ms, slice thickness (ST) 3 mm, in-plane resolution 0.3–0.5 mm); (ii) diffusion-weighted imaging (DWI) single-shot echo planar imaging (ssEPI) (b-values: 0,50, 500, 1000, 1500; TR 3300–5700 ms, TE 48–71 ms, ST 3 mm and in-plane resolution 2 mm) and (iii) DWI readout-segmented multi-shot echo planar imaging sequence (rsEPI; b-values: (0), 50, 1000; TR 4070–6260 ms, TE 45–54 ms, ST 3 mm and in-plane resolution 2 mm). Apparent diffusion coefficient (ADC) maps were calculated using the scanner’s vendor software. Originally, the scan protocol included only the ssEPI sequence; during the course of the inclusion period, the second DWI rsEPI sequence was added, resulting in fewer patients with both ssEPI and rsEPI.

### Segmentations and radiomics feature extraction

Prostate lesion segmentations for radiomics feature extraction were performed by two readers with 2 years (R1) and 1.5 (R2) years of experience in prostate mpMRI under supervision of an expert with 15 years of experience. First, R1 reviewed the clinical prostate MRI reports and the corresponding prostate lesion diagrams, and established side-by-side hanging protocols with the main and additional scan exams. All lesions called in the radiologist reports were marked with arrows; lesion detection was, thus, not part of the study. R1 then performed segmentations of the marked lesions in the main and additional scan exams, which were used for the inter-scan comparison. R2 performed segmentations of the main exams for inter-rater comparison. Later, after a washout-period (> 6 months), R1 performed a second segmentation of the main exams, allowing the intra-rater comparison. Segmentations were performed on the T2-weighted images and the ADC maps, and drawn to encompass the entirety of the prostate lesion in each sequence. The ADC segmentations were also used for the high-b value images as ADC maps are calculated thereof and scanner software performs co-registration automatically. For normalization to muscle tissue, R1 also drew a 3-dimensional region of interest in the left internal obturator muscle. The routine PACS viewing system - Centricity PACS Radiology RA1000 (GE Healthcare, Chicago, Illinois, USA) was used for evaluation of lesions, while the segmentations were performed using the Medical Imaging Toolkit software (MITK)^25^.

**Fig. 1:**
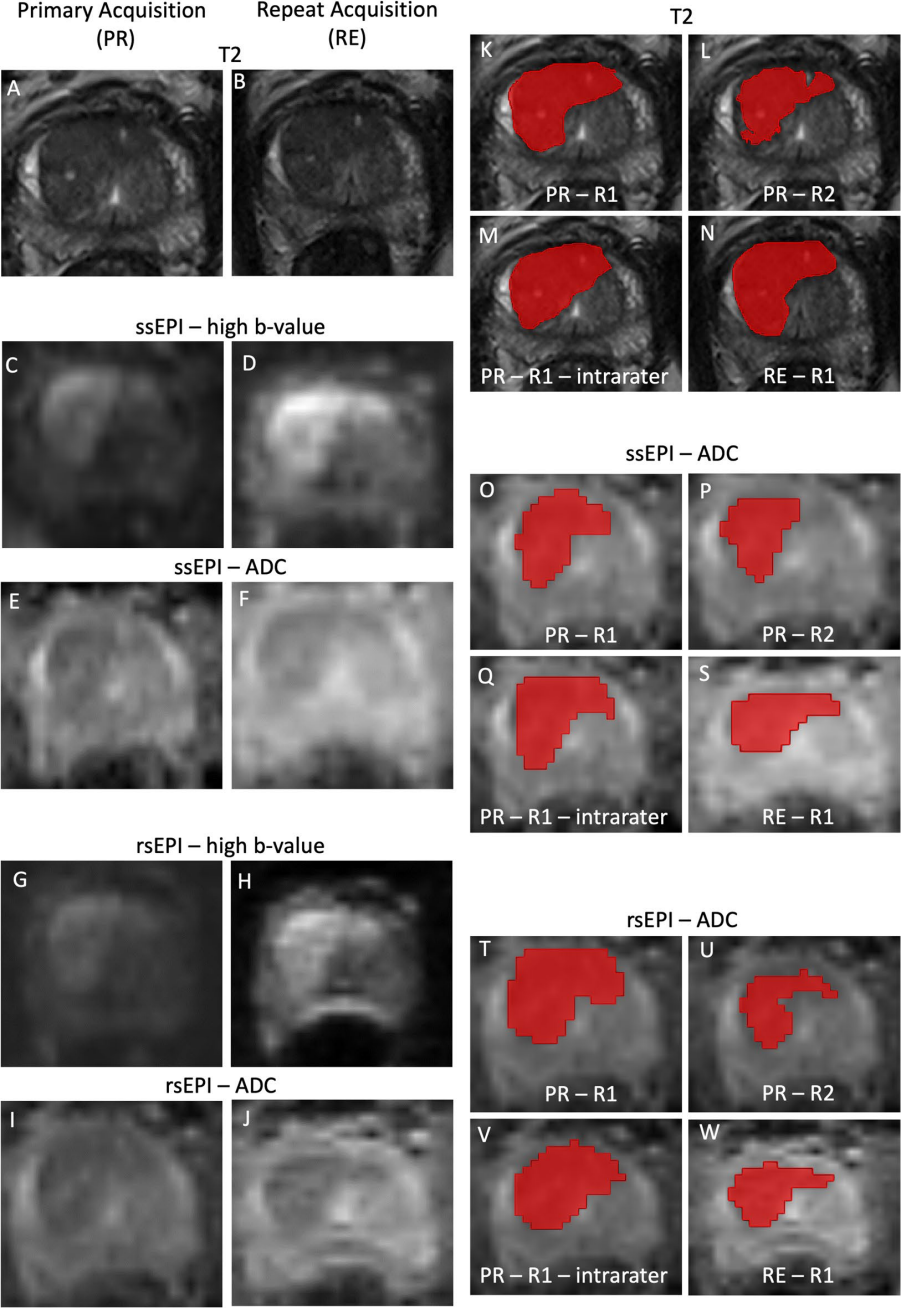
Visual example of a PIRADS 5 lesion in the middle anterior and posterior transition zone on the right side with extension over the centerline and corresponding segmentations. The patient was diagnosed with ISUP grade group 3 tumor at this location by targeted biopsy. (**A**,**B**) T2-weighted images, (**C**,**D**) ssEPI high-b-value images, (**E**,**F**) ssEPI calculated ADC maps, (**G**,**H**) rsEPI high-b-value images and (**I**,**J**) rsEPI calculated ADC maps for the primary and the repeat sequences, respectively. (**K**–**N**) demonstrate the T2-weighted images, (**O**–**S**) the ssEPI calculated ADC maps and (**T**–**W**) the rsEPI calculated ADC maps with the segmentations of Rater 1 and Rater 2, the intra-rater segmentation (R1) and the segmentation on the repeat sequences (R1), respectively. *PR* primary acquisition, *RE* repeat acquisition, *ssEPI* single-shot echo planar imaging, *rsEPI* readout-segmented multi-shot echo planar imaging, *R1/2* rater 1/2.

Feature Extraction was performed using PyRadiomics (version 3.1.0)^26^ in Python (version 3.8; Python Software Foundation, https://www.python.org/). PyRadiomics feature definitions, apart from few exceptions, adhere to the image biomarker standardization initiative (IBSI)^9^ and are given in the documentation (https://pyradiomics.readthedocs.io/en/v3.1.0/features.html). Standard settings for 3-dimensional extraction were used, resulting in the extraction of 107 features in total: shape-based (*n* = 14), first-order (*n* = 18), second-order: gray level co-occurrence matrix (GLCM, *n* = 24), gray level dependence matrix (GLDM, *n* = 14), gray level run length matrix (GLRLM, *n* = 16) gray level size zone matrix (GLSZM, *n* = 16) and neighboring gray tone difference matrix (NGTDM, *n* = 5). For the default list of enabled and disabled feature see the PyRadiomics documentation (https://pyradiomics.readthedocs.io/en/v3.1.0/features.html). For the complete list of extracted features see (Supplementary Table S1). To evaluate the variability of features under different normalization methods, T2WI and DWI sequence images were left as is, underwent Z-score or muscle normalization (to the left internal obturator muscle - LIOM): $\:{s}_{norm}=\frac{intensitie{s}_{rawImage}}{mean\:intensit{y}_{LIOM}}$. ADC maps, as a quantitative and calculated measurement, did not undergo normalization as previous studies have demonstrated good repeatability and prediction results for single-center studies without normalization^3,24,27^. To investigate benefit of N4B bias correction^28^ for intensity non-uniformities of MRI, features were extracted from T2WI before and after correction using SimpleITK for Python^29^. The IBSI expert panel recommends interpolation to isotropic voxels to enable rotational invariance and comparison between data from different samples, cohorts or batches^9^. However, the panel also states that up-sampling introduces artificial information not generated in the MRI measurement and down-sampling leads to information loss^9^. In addition, a considerable portion of features are sensitive to interpolation itself and even the interpolation algorithm affects feature stability^9,30^. However, our study considers a homogeneous single-scanner test/retest cohort with standardized positioning of patients for prostate MRI. The scan protocols between the test and retest image acquisitions were identical, i.e., identical voxel sizes, and test/retest analysis was only performed in the respective individual. Therefore, we opted against artificial voxel interpolation to avoid variability due to further processing steps, information loss by down-sampling or introduction of artificial information by up-sampling. Bin width for feature extraction was chosen to achieve a bin number of ~ 60–130 bins as IBSI recommends fixed bin numbers for discretization of MRI images^9^ and PyRadiomics supports this range for good reproducibility (https://pyradiomics.readthedocs.io/en/v3.1.0/faq.html); for exact bin widths of each sequence and corresponding normalization method, see supplementary table S2. A visual example of images generated in the test/retest setting and the segmentations performed by R1 and R2 are given in (Fig. 1). To assess agreement of the segmentations, dice coefficients were calculated: average coefficients were ~ 63% and ~ 53% for intra-rater and inter-rater comparisons, respectively (see supplementary Table S3).

### Statistical analysis

To evaluate stability of features, for each comparison, Lin’s concordance correlation coefficient (CCC) was estimated using variance components^31^ of a linear mixed model with patient as random effect to account for multiple lesions per patient^32^. Since the distribution of radiomics features was partially non-normal, a robust linear mixed model was fitted on the original (untransformed) feature data. The CCC values are mapped to the interval [−1,1], with perfect agreement at a value of 1^33^. A CCC threshold of 0.75 was defined as cut-off and features were considered ‘stable’ if the threshold was reached for all comparison.

Hierarchical cluster analysis was performed using all stable features and all prostate lesions possessing corresponding histopathology with sPC as ground truth. For clustering, feature values were standardized/scaled across lesions, and distance according to Pearson’s correlation coefficient and Ward’s linkage were used. sPC was defined as International Society of Urological Pathology (ISUP) grade group (GG) ≥ 2.

Random forest using 1000 trees with Boruta selection algorithm was used to identify relevant features for detection of sPC^34^. The Boruta algorithm creates random noise for each feature resulting in the ‘shadow feature’, which serve as baseline for importance comparison of the original ones. The output classifications of the Boruta algorithm are: ‘confirmed’ (more important that shadow features), ‘rejected’ (less important than shadow features) and ‘tentative’ (indeterminate between ‘confirmed’ and ‘rejected’). For further assessment, results of different selection algorithms, commonly used for feature selection, were added for comparison^35,36^: least absolute shrinkage (LASSO) algorithm^37^, minimum redundancy maximum relevance (MRMR) filter method^38^ and univariate analysis based on the Wilcoxon test. Statistical analysis was performed with software R (version 4.42) using add-on packages: lme4, robustlmm, pheatmap, Boruta, mRMRe and glmnet.

## Results

### Study sample

As previously described^24^between 2017 and 2021, 43 patients fulfilled the inclusion criteria for the final, prospective study. Detailed cohort information and an inclusion flow diagram have been published before^24^. In brief, patient characteristics were: (a) age – 66 years (IQR 60–69); (b) the maximum Prostate Imaging Reporting and Data System (PI-RADS) categories 1/2/3/4/5 for the patients were 0/2/17/17/7; (c) in total, 87 lesions of the 43 men were included for the study; (d) for *n* = 30 patients histology was available by systematic and targeted biopsy; 8 patients had no cancer, 11 non-significant cancer (nsPC) and 11 were diagnosed with significant prostate cancer (sPC); (e) on the lesional level 21 lesions possessed no histology, 36 lesions contained no cancer, 13 lesion nsPC and 17 lesions sPC, i.e., for 66 lesions of 30 patients histology was available. Thirty-three of the patients examined in this work have been part of training / test cohorts for retrospective evaluation of different machine learning systems, most recently^39,40^. These prior reports however used only the main scan data while the additional scan MR data have not been used in these reports.

Thirty-six patients received the additional scans before contrast agent administration and seven afterwards. All patients were pooled for the analysis, as no systematic bias could be detected as to whether the additional scan was performed before or after contrast agent application, both for ADC maps^24^ and for T2WI and DWI sequences, which is in agreement with the contrast mechanism of these sequences which does not emphasize the presence of contrast agent. Accounting for severe artefacts, missing sequences in the clinical image archive, the later addition of rsEPI to the scan protocol, main and repeat sequences were available (i) for 41 patients with 84 lesions for T2WI, (ii) for all 43 patients and all 87 lesions for ssEPI, (iii) while rsEPI was available for 37 patients with 73 lesions for the main and 28 patients with 54 lesions in the repeat scan. Consequently, inter-scan analysis was performed on patients/lesions 41/87 for T2WI, 43/87 for ssEPI, 28/54 for rsEPI; inter-sequence comparison between ssEPI and rsEPI on 37/73, respectively.

### Variability of radiomics features extracted from high b-value images of two DWI sequences

**Fig. 2:**
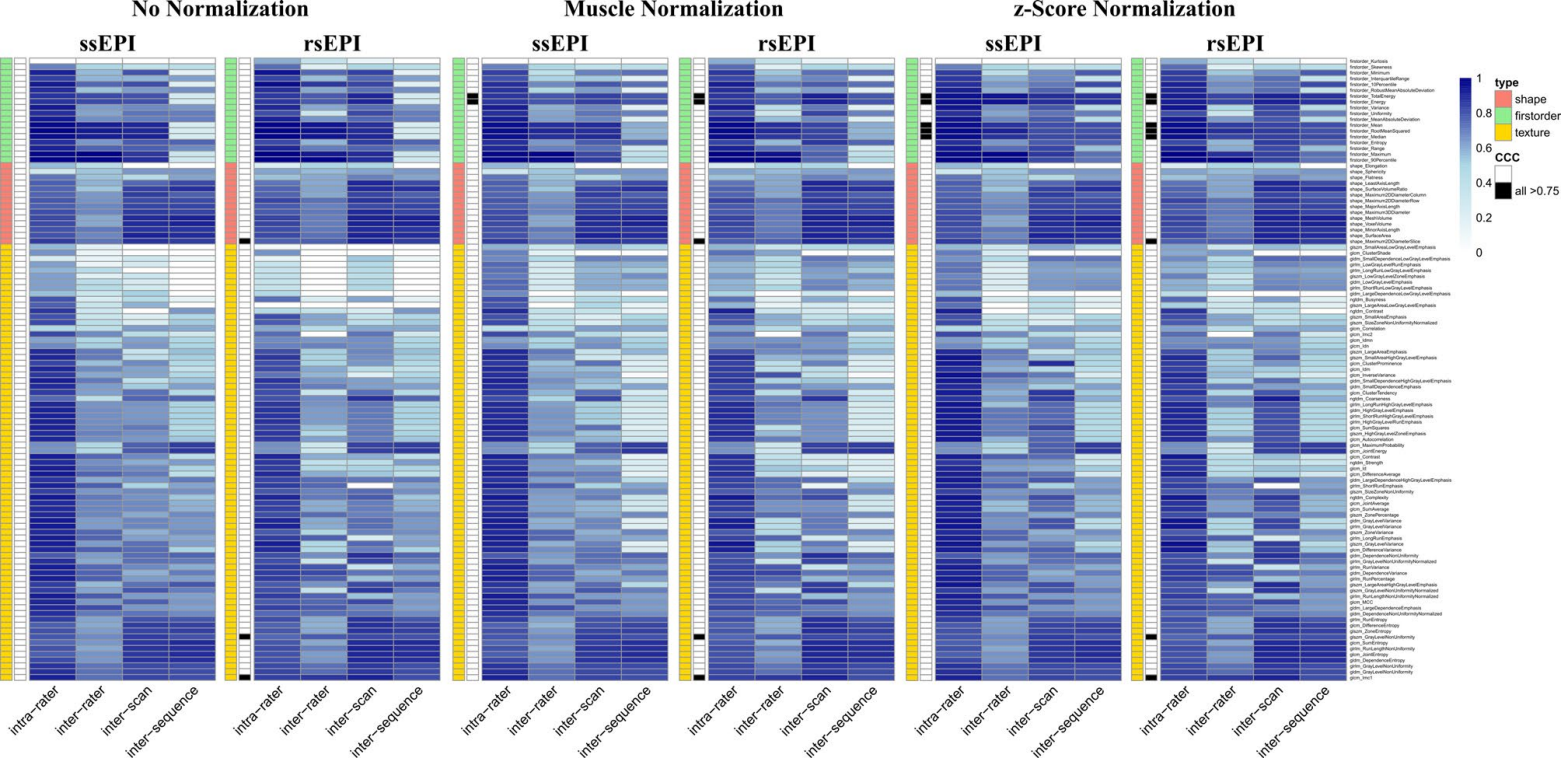
Graphical illustration of concordant correlation coefficients (CCC) for High-b value Images features of two diffusion-weighted imaging sequences for different variability comparisons (intra-rater, inter-rater/-scan/-sequence) under different normalization methods. Feature type (shape, first-order, texture) is color-coded. Features reaching a CCC ≥ 0.75 in all variability comparisons are marked in black. Generally, high-b value features demonstrate low stability for shape-features. Z-score normalization shows the highest number of features reaching CCC ≥ 0.75 with mainly first-order features. *ssEPI* single-shot echo planar imaging, *rsEPI* readout-segmented multi-shot echo planar imaging.

Comparing the two DWI sequences, rsEPI possessed slightly higher stability with 16 stable features across all normalization methods compared to 7 features for ssEPI (see Fig. 2). Second, normalization seemed to be essential for high-b value images of DWI; for both ssEPI and rsEPI, images with no normalization yielded the lowest number of stable features, while z-score normalization provided the highest rate. For both sequences, shape and second-order features were unreliable in most cases. With z-score normalization, five first-order features proved stability: firstorder_TotalEnergy, firstorder_Energy, firstorder_RootMeanSquared, firstorder_Median and firstorder_Mean. For visualization and detailed comparison, see (Fig. 2). For further, analyses the ssEPI sequence was chosen as it was the institutional standard DWI sequence with all 43 patients possessing test-retest data.

### Variability of radiomics features extracted from ADC maps of two DWI sequences

**Fig. 3:**
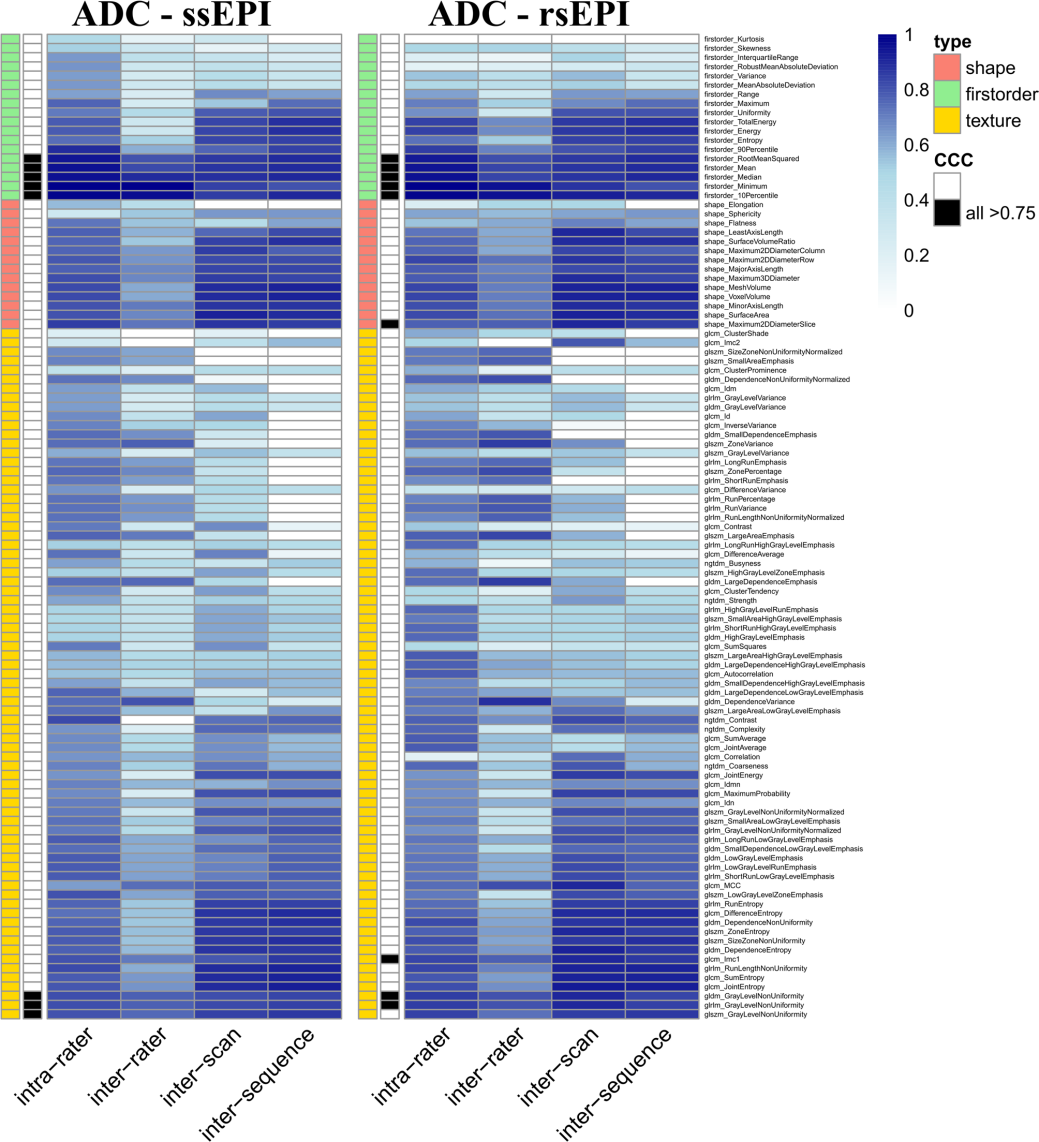
Graphical illustration of concordant correlation coefficients (CCC) for apparent diffusion coefficient (ADC) maps features calculated from two diffusion-weighted imaging sequences for different variability comparisons (intra-rater, inter-rater/-scan/-sequence). Feature type (shape, first-order, texture) is color-coded. Features reaching a CCC ≥ 0.75 in all variability comparisons are marked in black. As segmentations for high-b value images and ADC maps were identical, shape features were identical. Mainly first-order and few texture features reached the CCC stability cut-off. *ssEPI* single-shot echo planar imaging, *rsEPI* readout-segmented multi-shot echo planar imaging.

Generally, shape features seemed to lack stability for ADC maps. Of the first-order features, five were identified to possess stability: firstorder_RootMeanSquared, firstorder_Mean, firstorder_Median, firstorder_Minimum and firstorder_10Percentile. Two second-order features proved to be stable: glrlm_GrayLevelNonUniformity and gldm_GrayLevelNonUniformity. For visualization and detailed comparison, see (Fig. 3).

### Variability of radiomics features extracted from T2WI

**Fig. 4:**
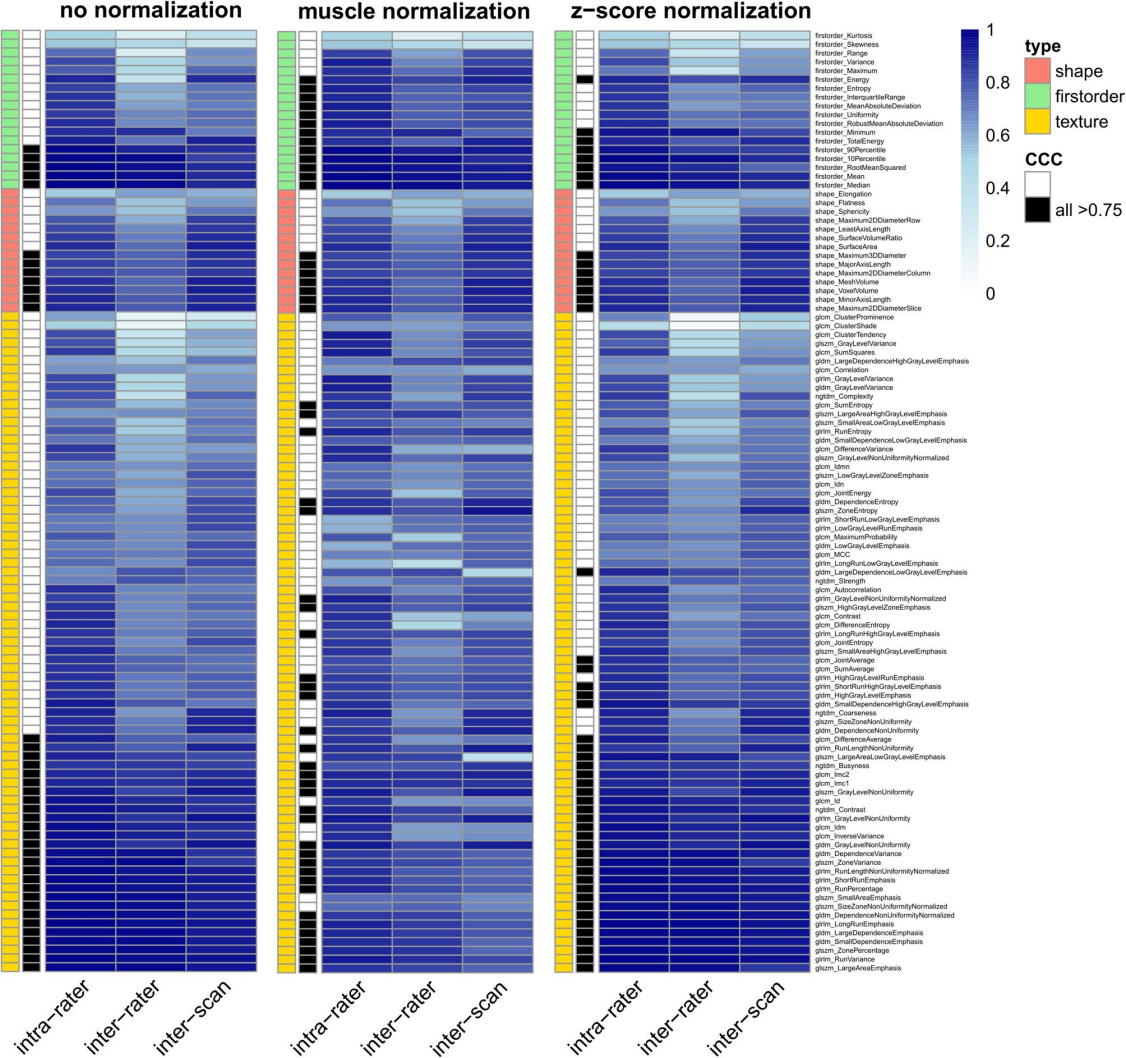
Graphical illustration of concordant correlation coefficients (CCC) for T2-weighted imaging (T2WI) features for different variability comparisons (intra-rater, inter-rater/-scan/-sequence) under different normalization methods. T2WI sequences were corrected for field inhomogeneity by N4 bias correction. Feature type (shape, first-order, texture) is color-coded. Features reaching a CCC ≥ 0.75 in all variability comparisons are marked in black. T2WI, as the anatomical sequence, demonstrated the highest amount of stable shape features, while simultaneously also the highest amount of texture features.

First, the effect of N4 bias correction was assessed: For both muscle and z-score normalization, bias correction increased the number of stable features from 42 to 52 and 43 to 46, respectively. For T2WI features without normalization, the number declined from 43 to 39. In summary, N4 bias correction seemed beneficial for feature stability. Muscle normalization after N4 bias correction resulted in the highest number of stable features (*n* = 52) and was chosen as the preferred normalization method. Generally, T2WI seemed to possess a higher number of stable features compared to DWI and the thereof calculated ADC maps, especially regarding shape features. However, it is worth noting that no inter-sequence comparison was available for T2WI. For visualization and detailed comparison, see Fig. 4; Table 1 for stable features with bias correction and Supplementary Figure S4 for features without bias correction.

**Table 1:** List of proposed stable features. High-b value features were z-score normalized and T2WI features were normalized to the internal obturator muscle signal intensity. ADC maps were not normalized due to their quantitative character.

High-b value features	T2WI features (part 2)
firstorder_TotalEnergy	glcm_Imc1
firstorder_Energy	glcm_Imc2
firstorder_RootMeanSquared	glcm_SumEntropy
firstorder_Median	gldm_DependenceEntropy
firstorder_Mean	gldm_DependenceNonUniformity
	gldm_DependenceNonUniformityNormalized
	gldm_DependenceVariance
**ADC Features**	gldm_GrayLevelNonUniformity
firstorder_RootMeanSquared	gldm_HighGrayLevelEmphasis
firstorder_Mean	gldm_LargeDependenceEmphasis
firstorder_Median	gldm_SmallDependenceEmphasis
firstorder_Minimum	glrlm_GrayLevelNonUniformity
firstorder_10Percentile.	glrlm_GrayLevelNonUniformityNormalized
glrlm_GrayLevelNonUniformity	glrlm_HighGrayLevelRunEmphasis
gldm_GrayLevelNonUniformity	glrlm_LongRunEmphasis
	glrlm_LongRunHighGrayLevelEmphasis
	glrlm_RunEntropy
**T2WI Features (part 1)**	glrlm_RunLengthNonUniformity
firstorder_10Percentile	glrlm_RunLengthNonUniformityNormalized
firstorder_90Percentile	glrlm_RunPercentage
firstorder_Energy	glrlm_RunVariance
firstorder_Entropy	glrlm_ShortRunEmphasis
firstorder_InterquartileRange	glrlm_ShortRunHighGrayLevelEmphasis
firstorder_MeanAbsoluteDeviation	glszm_GrayLevelNonUniformity
firstorder_Mean	glszm_HighGrayLevelZoneEmphasis
firstorder_Median	glszm_LargeAreaEmphasis
firstorder_Minimum	glszm_LargeAreaHighGrayLevelEmphasis
firstorder_RobustMeanAbsoluteDeviation	glszm_ZoneEntropy
firstorder_RootMeanSquared	glszm_Zone%
firstorder_TotalEnergy	glszm_ZoneVariance
firstorder_Uniformity	ngtdm_Busyness
	ngtdm_Contrast
shape_MajorAxisLength	
shape_Maximum2DDiameterColumn	
shape_Maximum2DDiameterSlice	
shape_Maximum3DDiameter	
shape_MeshVolume	
shape_MinorAxisLength	
shape_VoxelVolume	

In total, combining all sequences and accounting for the selected normalization methods, 64 features were deemed stable (five high b-value image features, seven ADC map features and 52 T2WI features). For the list of proposed stable features for all sequences see (Table 1).

Cluster analysis

For further evaluation, hierarchical cluster analysis was performed on the patients and lesions with available histology for all stable features from the three sequences (see Supplementary Figure S5). Three lesions of two patients were excluded from cluster analysis as T2WI sequences were missing or exhibited artefacts: one biopsy-negative and two sPC cancer lesions. The subset of stable features did possess a ‘weak’ clustering effect with regard to lesions containing and not containing sPC. A ‘positive’ clustering effect can be observed for the z-score normalized b-value image features firstorder_Median, firstorder_RootMeanSquared and firstorder_Mean, indicated in supplementary figure S5 by a red box, representing plausible predictors of sPC from the view of domain expert knowledge. In some cases clustering together, there were high values for T2- and ADC-derived NonUniformity and Energy features, which correspond to larger lesions judging by the related shape features, e.g., shape_Maximum3DDiameter and shape_MinorAxisLength.

Feature importance

**Fig. 5:**
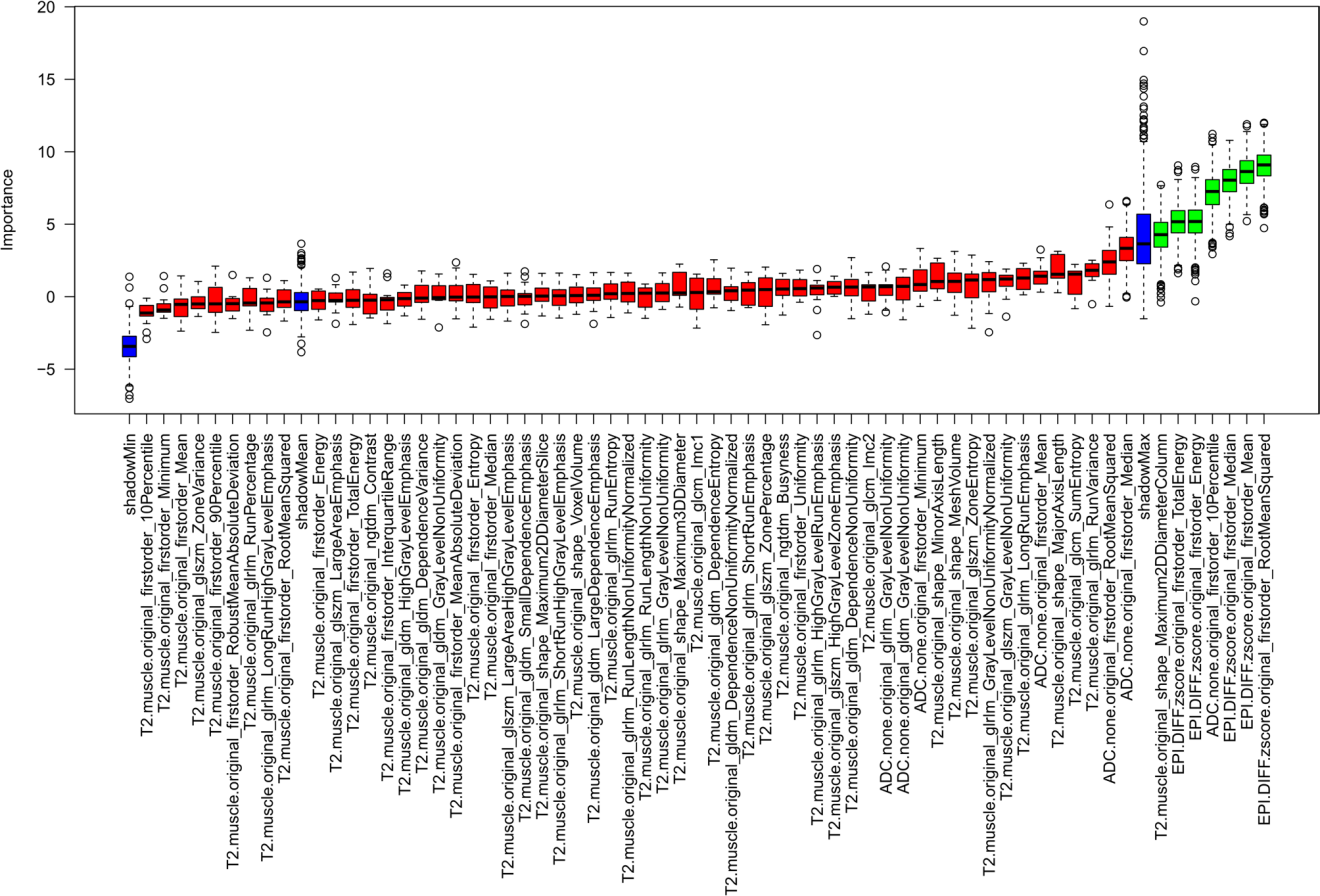
Output of the random-forrest based Boruta feature selection algorithm. Seven features were detemermined as important for prostate cancer identifcation by the Boruta algorithm (five high-b value, one ADC and one T2WI feature). *ADC* apparent diffusion coefficient, *T2WI* T2-weighted imaging.

**Fig. 6:**
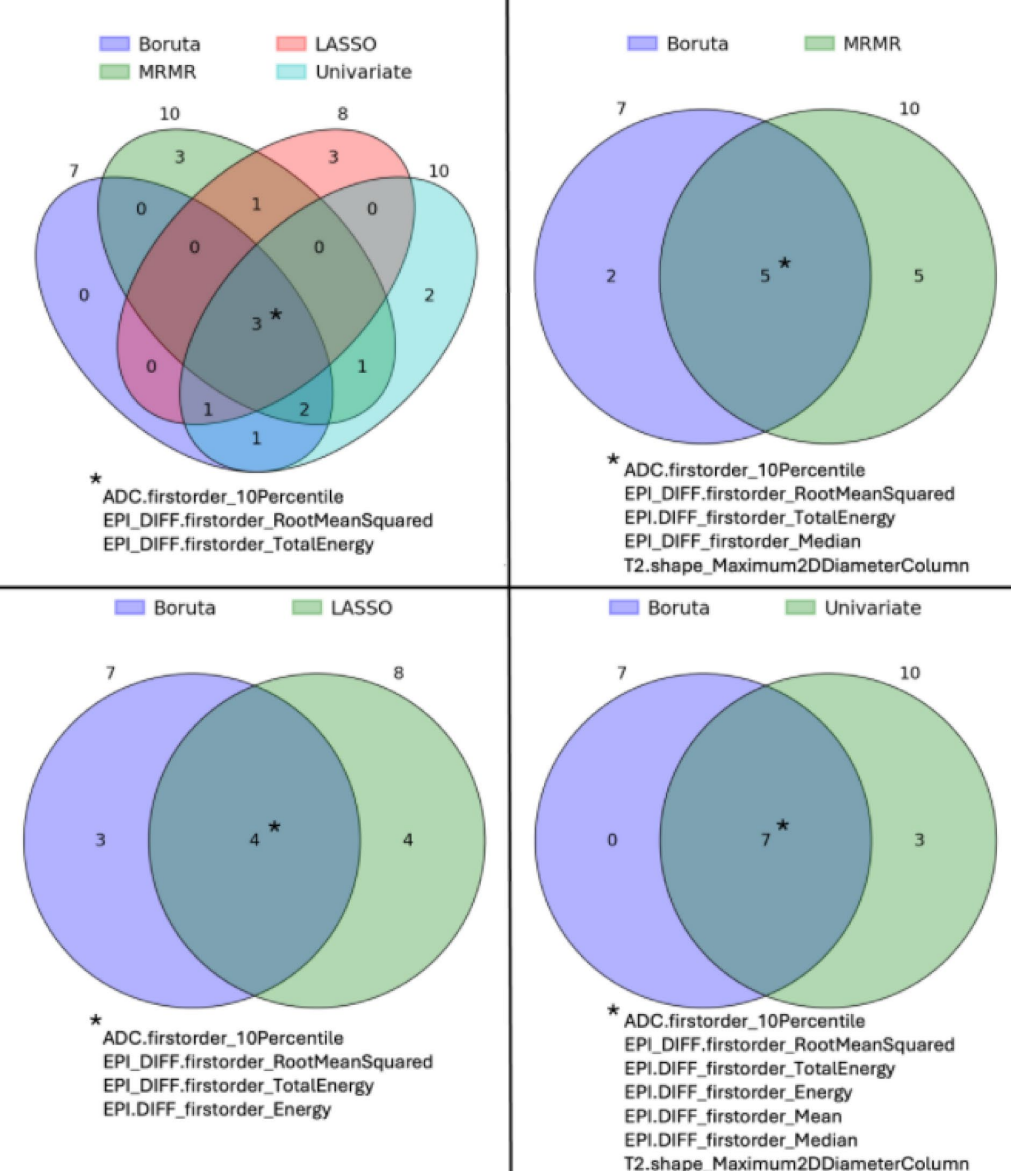
Comparison of different radiomics feature selection methods using Venn diagrams: Boruta, minimum redundancy maximum relevance (MRMR), least absolute shrinkage (LASSO) and Univariate screening algorithms. Venn diagrams demonstrate the overlap of the different feature selection methods: Boruta algorithm (wrapper method); LASSO algorithm (embedded method), MRMR algorithm (filter method) and screening using univariate analysis with presence of significant prostate cancer as the prediction / dependent variable. Boruta algorithm selected features possessed with considerable overlap features selected by other methods. Three features were considered important by all: ADC based firstorder_10Percentile, and firstorder_RootMeanSquared and firstorder_TotalEnergy from z-score normalized high b-value images. *MRMR* minimum redundancy maximum relevance, *LASSO* least absolute shrinkage.

To select features with predictive performance regarding prostate cancer detection, different feature selection algorithms were used upon all stable features. The Boruta feature selection identified seven important features (see Fig. 5; Table 2): 5 high b-value features, 1 T2WI shape feature and the ADC-based firstorder_10Percentile feature. The embedded feature selection method LASSO classified 8 features as important: 4 T2WI texture features, 3 high b-value features and one ADC feature (Table 2). The top 10 features of both the MRMR method and the univariate analysis are given in (Table 2). All feature selection methods showed considerable overlap with the Boruta selection. Venn diagrams for all selection methods and the overlaps between the Boruta algorithm and the other methods are given in (Fig. 6). Three features were consistently chosen by all selection methods: ADC-based firstorder_10Percentile feature and the z-score normalized high b-value features: firstorder_TotalEnergy, firstorder_RootMeanSquared.

**Table 2:** List of features selected by different feature selection methods/algorithms: random forest-based Boruta selection, LASSO (least absolute shrinkage) analysis, MRMR (minimum redundancy maximum relevance) algorithm and univariate analysis. High-b value features were z-score normalized and T2WI features were normalized to the internal obturator muscle signal intensity. ADC maps were not normalized due to their quantitative character.

Boruta	LASSO
ADC.firstorder_10Percentile	T2.glcm_SumEntropy
EPI_DIFF.firstorder_Mean	T2.gldm_DependenceEntropy
EPI_DIFF.firstorder_Median	T2.glrlm_LongRunHighGrayLevelEmphasis
EPI_DIFF.firstorder_RootMeanSquared	T2.glszm_LargeAreaHighGrayLevelEmphasis
EPI_DIFF.firstorder_Energy	EPI_DIFF.firstorder_TotalEnergy
EPI_DIFF.firstorder_TotalEnergy	EPI_DIFF.firstorder_Energy
T2.shape_Maximum2DDiameterColumn	EPI_DIFF.firstorder_RootMeanSquared
	ADC.none.original_firstorder_10Percentile

MRMR	Univariate analysis
EPI_DIFF.firstorder_RootMeanSquared	EPI_DIFF.firstorder_RootMeanSquared
ADC.firstorder_10Percentile	EPI_DIFF.firstorder_TotalEnergy
T2.shape_Maximum2DDiameterColumn	EPI_DIFF.firstorder_Energy
EPI_DIFF.firstorder_TotalEnergy	EPI_DIFF.firstorder_Mean
T2.glcm_SumEntropy	EPI_DIFF.firstorder_Median
T2.glrlm_RunVariance	ADC.firstorder_10Percentile
ADC.firstorder_Minimum	ADC.firstorder_Mean
T2.firstorder_Minimum	ADC.firstorder_RootMeanSquared
ADC.firstorder_Median	ADC.firstorder_Median
EPI_DIFF.firstorder_Median	T2.shape_Maximum2DDiameterColumn

## Discussion

Despite great success in academic research, the translation of promising research results in radiomics into radiologic practice has thus far been insufficient to non-existent. A major factor contributing to this phenomenon is the question of generalizability. According to different expert panels, the isolation of a stable (sub-)set of radiomics features might be a further step for the clinical translation of radiomic machine learning models^8,17,18^.

In accordance with previous studies^11,16^our results demonstrated that only a fraction of extracted radiomics features proved to be stable under varying conditions: in this case 64 of total extracted 321 (~ 20%) features proved intra-/inter-rater, inter-sequence and inter-scan stability. With further feature selection, even fewer features remained. We performed exploratory analyses using different feature selection algorithms (non-linear: Boruta, MRMR; linear: LASSO, univariate analysis). Boruta was considered the main selection algorithm, as this wrapper method built around random forests is considered robust, less prone to overfitting, provides an ‘all-relevant’ subset, captures high-dimensional data and, therefore, is often used as a selection algorithm in radiomics studies^34–36,41^and as these advantages are especially important in small sample sizes. As expected, the different selection methods did not yield identical results due to their different dimensionality and their main concept, e.g. MRMR is designed to not only check for relevance but also for redundancy, thus, reducing the feature set in size^38^. Boruta demonstrated considerable overlap with the other methods, and all features chosen by Boruta were among the top 10 of the features ranked by univariate analysis, supporting the validity of the Boruta results. Currently, there is no consensus on the choice of selection algorithms^35,42^ and, considering the cohort size, the results of the different analyses should be considered exploratory.

For the high-b value DWI sequences, z-score normalization on the whole image resulted in the highest number of stable features. For ADC images, no normalization approach was used, as ADC itself is a quantitatively calculated parameter and previous studies have demonstrated good prediction results for single-center studies and repeatability^3,24,27^. It was prominent that for high-b value and ADC images, the shape features demonstrated low stability, which might be expected as DWI is not considered an anatomical sequence but instead a functional MRI technique based on lower resolution images with larger voxel size^43^. In a short-term repeatability study, Merisaari et al. demonstrated stability of shape features in DWI, however, their study excluded lesions with a diameter ≤ 5 mm and there was no reproducibility analysis for inter-rater variation^23^. The three features identified by all selection algorithms were derived from high b-value or ADC images, underscoring the importance of DWI in prostate MRI, which is in accordance with the PI-RADS classification and previous studies: according to Bonekamp et al. 7 of the 10 most important features were DWI-based^3^while the radiomics signature of Bao et al. in their retrospective multi-center study contained 25 features, 17 of which were DWI-derived^44^.

While DWI sequences are considered ‘functional’, T2WI sequences are less susceptible to imaging artefacts and used for anatomy and morphological assessment of the prostate and its lesions utilizing much smaller voxel sizes. This would suggest a larger number of stable shape features, as was demonstrated by our results. Another noticeable finding, which might be related to the different voxel sizes, is the higher number of stable texture features for T2WI. Due to their high-resolution, T2WI segmentations contain more voxels, which may attenuate the effect of segmentation variability on texture features. It is worth noting that, compared to the DWI analysis, no inter-sequence analysis was available for T2WI sequences, which may also increase the number of stable shape and texture features.

The importance of T2WI texture-derived features remains to be debated: Some studies demonstrated benefit for prostate cancer prediction tasks^45,46^however, the Boruta algorithm of our study considered only one feature important. Accordingly, some previous studies support only limited predictive value of T2WI features: Wibmer et al.^47^ demonstrated no association of T2-derived texture features with Gleason score and Bonekamp et al. showed that among the 10 most important features of their random-forest based model no T2WI-texture feature was included^3^. The results of our study allow no definite statement concerning the preferred normalization method for T2WI sequences. All normalization methods (none, muscle and z-score) provided a relatively high number of stable features. Our results demonstrated that the stability of T2WI features was variable depending on the normalization method, which is in accordance to the results of Schwier et al., who showed that with muscle normalization the stability of T2WI features might in- or decrease^16^.

To our knowledge, this study comprises the largest prostate MRI test/retest cohort containing both T2WI and (two) DWI sequences with 43 patients. In general, prospective test/retest studies are rare, possibly due to increased radiation exposure with CT and limited/expensive scanner time with MRI. Schwier et al. performed one of the first comprehensive radiomics feature stability studies, analyzing different normalization and filter methods, and evaluating different bin widths during extraction, however, their study did not consider variability due to inter-rater effects or different sequences, i.e., inter-sequence effects, and their dataset included only 15 patients (two MRIs within two weeks)^16^; furthermore, b-value images were not part of the analysis. Merisaari et al. evaluated a comparably large collective of 112 patients undergoing repeat DWI scans on the same day; analysis was performed only on ADC and kurtosis maps, while intra- and inter-rater effects were not considered^23^. Interestingly, Merisaari et al. reported high repeatability for the shape feature SurfaceVolumeRatio, which in our analysis was eliminated due to lack of inter-rater stability. Our study expands on the existing body of knowledge in prostate radiomics by accounting for variability due to repositioning, rater effects and different DWI sequences, and evaluating different normalization methods. Despite several expert panels considering the isolation of stable radiomics features as beneficial for the clinical translation of radiomic machine learning models^8,17,18, ^a recent study demonstrated that using only reproducible radiomics features instead of all features does not necessarily improve the performance and generalizability of radiomics models for multicentric application^48^. Subsequent studies should investigate whether the identified features in our study can improve the predictive performance of radiomics models for prostate cancer.

There are limitations to this study. First, the single center, single scanner design may limit generalizability of identified stable features to different field strengths^11,12, ^other vendors and especially external multi-centric datasets. However, the homogeneous setup enables identification of largest subset potentially useful features, while eliminating those susceptible to rater or positioning effects. Second, the detection rate of prostate MRI itself is limited, as ca. 10% of sPC are not identified on MRI^49, ^which makes lesion-based radiomics feature extraction unfeasible. Third, the dataset of 43 patients with 30 of these possessing histopathological reference is limited, although it is the largest prostate MRI test/retest cohort containing both T2WI and DWI sequences^16,23,50,51^. In this context, inter-sequence comparison was partially limited as it was introduced later during the study period resulting in less patients with both ssEPI and rsEPI. Further, the histopathology-guided feature selection by the different algorithm may be inaccurate due to the limited number of patients and sPC lesions. It is established that larger training sets improve performance and generalizability of machine learning models, and are therefore desirable. However, Thian et al. have demonstrated a ceiling effect or ‘diminishing performance returns’ once the training data have reached a certain amount^52^. Therefore, unlimited training data acquisition is not sensible, instead, a cost-benefit ratio must be kept in mind while increasing data. ComBat harmonization has been proposed for normalization of radiomics feature extracted from different sites. As this study was a single-scanner study, the method was not investigated.

### Conclusion

This study presents a single-center investigation of radiomics feature stability in prostate MRI, addressing a critical gap in reproducibility research. While limitations in sample size and generalizability exist, the findings provide valuable insights for feature selection and normalization protocols, serving as a foundation for future multi-center validation. The extracted features may contribute to establishing a stable radiomics signature to aid in the translation of radiomics from research to clinical practice.

## Electronic supplementary material

Below is the link to the electronic supplementary material.

Supplementary Material 1

## Data Availability

The datasets generated during and/or analysed during the current study are not publicly available due to national data protection laws but are available from the corresponding author on reasonable request after approval by the institutional data protection office.
