# Supplementary material for: In vivo variability of MRI radiomics features in prostate lesions assessed by a test-retest study with repositioning
Source: Sci Rep. 2025 Aug 13;15:29703. doi: 10.1038/s41598-025-09989-7 (PMC12350850; doi:10.1038/s41598-025-09989-7)

**Supplementary Material**

**Supplementary Table S1. Features Extracted Using PyRadiomics (v3.0)**

| **Shape Features** | **First-Order Features** |
| --- | --- |
| Elongation | 10Percentile |
| Flatness | 90Percentile |
| LeastAxisLength | Energy |
| MajorAxisLength | Entropy |
| Maximum2DDiameterColumn | InterquartileRange |
| Maximum2DDiameterRow | Kurtosis |
| Maximum2DDiameterSlice | Maximum |
| Maximum3DDiameter | MeanAbsoluteDeviation |
| MeshVolume | Mean |
| MinorAxisLength | Median |
| Sphericity | Minimum |
| SurfaceArea | Range |
| SurfaceVolumeRatio | RobustMeanAbsoluteDeviation |
| VoxelVolume | RootMeanSquared |
|  | Skewness |
|  | TotalEnergy |
|  | Uniformity |
|  | Variance |
|  |  |
| **Second-Order Features** |  |
| **GLCM** | **GLDM** |
| Autocorrelation | DependenceEntropy |
| ClusterProminence | DependenceNonUniformity |
| ClusterShade | DependenceNonUniformityNormalized |
| ClusterTendency | DependenceVariance |
| Contrast | GrayLevelNonUniformity |
| Correlation | GrayLevelVariance |
| DifferenceAverage | HighGrayLevelEmphasis |
| DifferenceEntropy | LargeDependenceEmphasis |
| DifferenceVariance | LargeDependenceHighGrayLevelEmphasis |
| Id | LargeDependenceLowGrayLevelEmphasis |
| Idm | LowGrayLevelEmphasis |
| Idmn | SmallDependenceEmphasis |
| Idn | SmallDependenceHighGrayLevelEmphasis |
| Imc1 | SmallDependenceLowGrayLevelEmphasis |
| Imc2 |  |
| InverseVariance |  |
| JointAverage | **NGTDM** |
| JointEnergy | Busyness |
| JointEntropy | Coarseness |
| MCC | Complexity |
| MaximumProbability | Contrast |
| SumAverage | Strength |
| SumEntropy |  |
| SumSquares |  |
|  |  |
| **GLRM** | **GLSZM** |
| GrayLevelNonUniformity | GrayLevelNonUniformity |
| GrayLevelNonUniformityNormalized | GrayLevelNonUniformityNormalized |
| GrayLevelVariance | GrayLevelVariance |
| HighGrayLevelRunEmphasis | HighGrayLevelZoneEmphasis |
| LongRunEmphasis | LargeAreaEmphasis |
| LongRunHighGrayLevelEmphasis | LargeAreaHighGrayLevelEmphasis |
| LongRunLowGrayLevelEmphasis | LargeAreaLowGrayLevelEmphasis |
| LowGrayLevelRunEmphasis | LowGrayLevelZoneEmphasis |
| RunEntropy | SizeZoneNonUniformity |
| RunLengthNonUniformity | SizeZoneNonUniformityNormalized |
| RunLengthNonUniformityNormalized | SmallAreaEmphasis |
| RunPercentage | SmallAreaHighGrayLevelEmphasis |
| RunVariance | SmallAreaLowGrayLevelEmphasis |
| ShortRunEmphasis | ZoneEntropy |
| ShortRunHighGrayLevelEmphasis | ZonePercentage |
| ShortRunLowGrayLevelEmphasis | ZoneVariance |

**Supplementary Table S2. Signal Intensity (SI) Metrics and Bin widths used for Feature Extraction**

|  | **t2**  **none** | **t2**  **zscore** | **t2**  **muscle** | **epi_b**  **none** | **epi_b**  **zscore** | **epi_b**  **muscle** | **epi_adc**  **none** | **resolve_b**  **none** | **resolve_b**  **zscore** | **resolve_b**  **muscle** | **resolve_adc**  **none** |
| --- | --- | --- | --- | --- | --- | --- | --- | --- | --- | --- | --- |
| SI Median | 323,50 | 2,25 | 7,49 | 15,00 | 3,85 | 1,45 | 538,00 | 17,00 | 3,91 | 0,94 | 554,00 |
| SI Mean | 332,93 | 2,26 | 8,10 | 16,09 | 4,91 | 1,61 | 578,69 | 18,08 | 4,05 | 1,00 | 602,15 |
| SI SD | 111,67 | 0,70 | 3,46 | 7,61 | 2,94 | 0,80 | 236,96 | 8,93 | 2,09 | 0,54 | 277,12 |
| SI MAX | 620,00 | 4,06 | 21,80 | 41,00 | 16,52 | 4,87 | 1327,00 | 59,00 | 11,53 | 3,50 | 1627,00 |
| SI MIN | 95,00 | 0,70 | 2,32 | 5,00 | 1,11 | 0,54 | 205,00 | 4,00 | 0,52 | 0,15 | 115,00 |
| Bin size | 5 | 0,05 | 0,2 | 0,4 | 0,1 | 0,04 | 12 | 0,4 | 0,1 | 0,04 | 12 |
| Number of est. bins | 105 | 67 | 97 | 90 | 154 | 108 | 94 | 138 | 110 | 84 | 126 |

Approach: First, the mean, median, standard deviation, maximum and minimum were extracted from the signal intensities of the volumes of the respective MRI images within the segmentation volumes of R1. Then, different bin sizes were tested manually for each sequence and normalization method separately as to obtain a bin size, for which the quotient of the range (SI_MAX – SI_MIN) and the bin size would result in a number of bins between the recommended 30 – 130 bins:

$$\frac{range(SI_{MAX}-SI_{MIN})}{bin widt} \sim30-130 bins$$

Rationale: IBSI recommends fixed bin numbers for discretization of MRI images and PyRadiomics supports this range for good reproducibility of radiomics features (https://pyradiomics.readthedocs.io/en/v3.1.0/faq.html).

Abbreviations: SD, standard deviation; t2, T2-weighted images; epi_b, high b-value images of the single-shot echo planar imaging diffusion sequence; epi_adc, ADC-map calculated from the single-shot echo planar imaging diffusions sequence; resolve_b, high b-value images of the readout-segmented multi-shot echo planar imaging diffusion sequence; resolve_adc, ADC map calculated from the readout-segmented multi-shot echo planar imaging diffusion sequence

**Supplementary Table S3. Dice Coefficients for comparison of segmentations for intra-rater (R1) and inter-rater (R1/R2) analysis.**

|  | T2WI | | ssEPI | | rsEPI | |
| --- | --- | --- | --- | --- | --- | --- |
|  | Intra-rater | Inter-rater | Intra-rater | Inter-rater | Intra-rater | Inter-rater |
| 95% percentile | 0,85 | 0,80 | 0,89 | 0,85 | 0,84 | 0,82 |
| 5% percentile | 0,31 | 0,08 | 0,27 | 0,00 | 0,12 | 0,13 |
| mean | 0,64 | 0,54 | 0,64 | 0,53 | 0,60 | 0,52 |
| median | 0,66 | 0,59 | 0,67 | 0,57 | 0,67 | 0,55 |

Abbreviations: ssEPI, single-shot echo planar imaging; rsEPI, readout-segmented multi-shot echo planar imaging; T2WI, T2-weighted imaging

**Supplementary Figure S4. Stable T2WI Features (Without previous N4 Bias Correction of Images)**


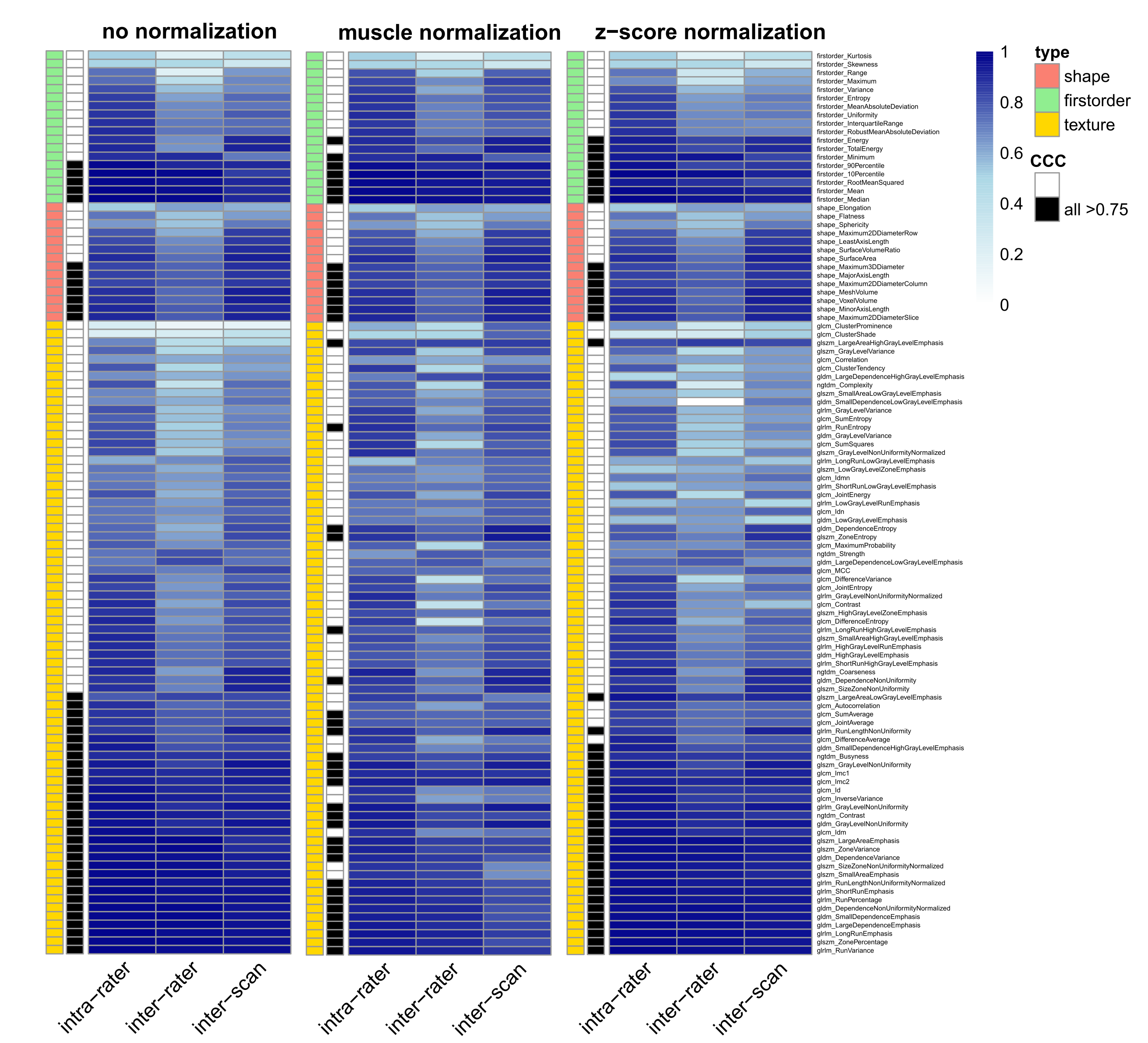


**Supplementary Figure S5. Two-dimensional hierarchical clustering heatmap generated from stable features with clinically significant prostate cancer (sPC) as ground truth.**

Features were taken from muscle-normalized T2WI, z-score normalized high-b value features and ADC maps. Feature column scaling was performed. Only lesions from patients with histopathological reference were included. A weak clustering effect was demonstrated for the high-b value firstorder features: Median, RootMeanSquared and Mean, especially in the lower rows.


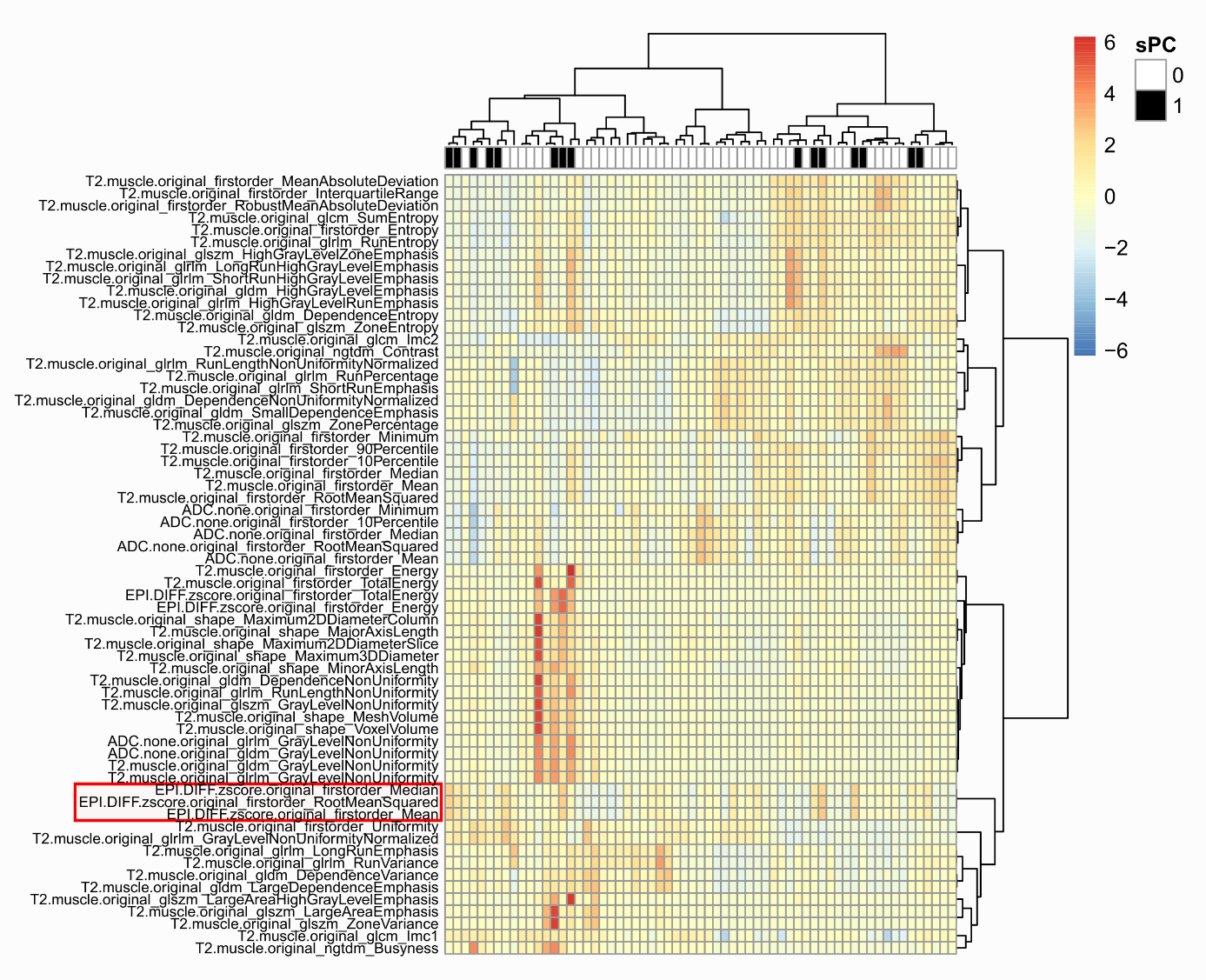

Supplement: Supplementary file 1 — Supplementary Material 1 [file 41598_2025_9989_MOESM1_ESM.docx]
